# Computational and experimental assessment of influences of hemodynamic shear stress on carotid plaque

**DOI:** 10.1186/s12938-017-0386-z

**Published:** 2017-07-29

**Authors:** Hui Zhou, Long Meng, Wei Zhou, Lin Xin, Xiangxiang Xia, Shuai Li, Hairong Zheng, Lili Niu

**Affiliations:** 1 Paul C. Lauterbur Research Center for Biomedical Imaging, Institute of Biomedical and Health Engineering, Shenzhen Institutes of Advanced Technology, Chinese Academy of Sciences, 1068 Xueyuan Ave., Nanshan District, Shenzhen, 518055 People’s Republic of China; 2 Shenzhen College of Advanced Technology, University of Chinese Academy of Sciences, Beijing, 100049 People’s Republic of China; 3 Department of Cardiology, Guangzhou General Hospital of Guangzhou Military Region, PLA, Guangzhou, 510010 People’s Republic of China

**Keywords:** Hemodynamic shear stress, Computational fluid dynamics, Ultrasonic particle imaging velocimetry, Atherosclerosis, Plaque

## Abstract

**Background:**

Studies have identified hemodynamic shear stress as an important determinant of endothelial function and atherosclerosis. In this study, we assess the influences of hemodynamic shear stress on carotid plaques.

**Methods:**

Carotid stenosis phantoms with three severity (30, 50, 70%) were made from 10% polyvinyl alcohol (PVA) cryogel. The phantoms were placed in a pulsatile flow loop with the same systolic/diastolic phase (35/65) and inlet flow rate (16 L/h). Ultrasonic particle imaging velocimetry (Echo PIV) and computational fluid dynamics (CFD) were used to calculate the velocity profile and shear stress distribution in the carotid stenosis phantoms. Inlet/outlet boundary conditions used in CFD were extracted from Echo PIV experiments to make sure that the results were comparable.

**Results:**

Echo PIV and CFD results showed that velocity was largest in 70% than those in 30 and 50% at peak systole. Echo PIV results indicated that shear stress was larger in the upper wall and the surface of plaque than in the center of vessel. CFD results demonstrated that wall shear stress in the upstream was larger than in downstream of plaque. There was no significant difference in average velocity obtained by CFD and Echo PIV in 30% (p = 0.25). Velocities measured by CFD in 50% (93.01 cm/s) and in 70% (115.07 cm/s) were larger than those by Echo PIV in 50% (60.26 ± 5.36 cm/s) and in 70% (89.11 ± 7.21 cm/s).

**Conclusions:**

The results suggested that Echo PIV and CFD could obtain hemodynamic shear stress on carotid plaques. Higher WSS occurred in narrower arteries, and the shoulder of plaque bore higher WSS than in bottom part.

## Background

Vulnerable plaque is considered to be the culprit of vessel thrombosis and acute cardiovascular and cerebrovascular diseases because of its silent progression and sudden rupture. Therefore, finding accurate and early predictor of plaque prone to rupture is essential for prospective and preventative treatment. A large amount of clinical studies have confirmed that vulnerable plaque is made of a thin overlying fibrous cap, large lipid cores and potential inflammation [1, 2]. Imaging technique can detect the existence of atherosclerotic plaque [3–5]. Intravascular ultrasound (IVUS) and optical coherence tomography (OCT) provide intravascular imaging and identify thin fibrous cap which is responsible for high probability rupture plaque [6, 7]. However IVUS and OCT are invasive tools. Magnetic resonance imaging (MRI) is a noninvasive technique which offers important biological characteristics of plaque [8], but its temporal-spatial resolution is low.

In addition, many studies have indicated that hemodynamic stress, such as wall shear stress (WSS) [9, 10], oscillatory shear index (OSI) [11], stress phase angle (SPA) [12], wall shear gradient (WSG) [13] has potential function in prediction of high-risk plaque. Among all those stress, WSS has been most fully studied. WSS affected the morphology and biochemistry of endothelial cells (ECs) and further influenced arterial remodelling and process of atherosclerosis [14, 15]. When healthy arteries were in high WSS (15–70 dyne/cm^2^), ECs present elongated arrangement parallel to the direction of flow, and high WSS will cause expression of vasodilators [16], fibrinolytics [17], in addition to reduce expression of leukocyte adhesion molecules [18] and inflammatory mediators [19], thus leading to expansive remodelling in the vessel to reduce WSS [20]. Whereas low WSS (<10 dyne/cm^2^) will decrease production of vasodilators [21], fibrinolytics [17], and increase expression of cell adhesion molecules [22], growth factors [23], thus leading to narrowing remodelling in the vessel to enhance WSS [20]. However, higher WSS may promote plaque rupture in artery stenosis.

Echo PIV, a two-dimensional, non-invasive ultrasonic velocimetry technique, could calculate the displacements of particles in fluid using cross-correlation coefficient, and the displacements of particles reflect fluid flow information. It has been confirmed that Echo PIV used in this research can conduct rotating flow, jet flow, tube flow measurement accurately [24, 25]. CFD, numerical computational method, has been widely used to calculate hemodynamic stress [26–31] in patient-specific images model [32, 33] and anatomically realistic experimental model [34].

The goal of this work is to conduct Echo PIV in three different severity stenosis PVA-c arterial phantoms, to obtain the hemodynamic shear stress in those phantoms and further analyze the influence of severity on the plaque. Besides, Echo PIV-based phantoms and boundaries were used in CFD to calculate WSS in three phantoms, and further verify the stenosis influence in plaque rupture.

## Methods

### PVA-c arterial phantoms

**Fig. 1:**
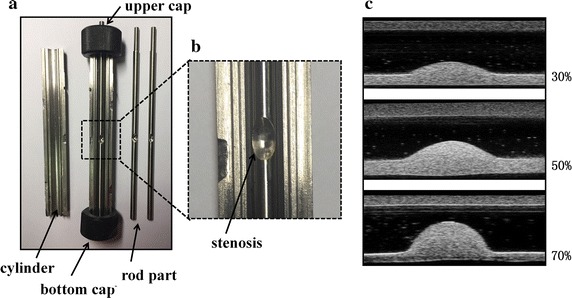
Alloyed mold used to manufacture carotid phantoms. **a** The cutaway view of the mold, containing: a two-pair cylinder (inner diameter 7 mm), a rod part (diameter 5 mm) and two caps to fix cylinder. **b** Enlarged view of the stenosis. **c** Ultrasound images of three stenosis phantoms

The carotid stenosis phantoms with three severity (30, 50, 70%) were made in this study. These phantoms had the same inner diameter (5 mm), wall thickness (1 mm), and length of plaque (8 mm). Polyvinyl alcohol (PVA) cryogel, which can mimic the behavior of arterial tissue and be an ideal material for ultrasound imaging phantoms [35, 36], was selected in this study. PVA solutions were prepared by mixing PVA power (Sigma-Aldrich Co, USA) in distilled water at room temperature (wt 10%:87%). The mixture was heated and shaken to make sure that PVA powder were fully dissolved. The mixture container was kept sealed during heating process to avoid drying. When the resulting clear solution was cooled to room temperature, wt 3% cellulose (Sigma-Aldrich Co, USA) was put into the solution, to allow for visualization of artery phantom under ultrasound system. Alloyed mold (Fig. 1a), which consisted of a two-pair cylinder (inner diameter 7 mm), and a rod part (diameter 5 mm) with progressively decreasing diameters at the midlength (Fig. 1b) to shape the stenosis with upper/bottom caps to fix the rod part, was used to contain the mixed solution and fabricate the phantoms. The mixed solution was poured into the mold with the rod part in it, and slowly to avoid bubbles. In order to form gel, the mold full with solution was submitted to repeated thermal and freezing cycles (12:12 h) from +20 °C to −4 °C. Gel stiffness depends on the number of thermal and freezing cycles [37]. In this study the gel went through 7 cycles. The ultrasound images for three phantom were shown in Fig. 1c.

### Experimental setup

**Fig. 2:**
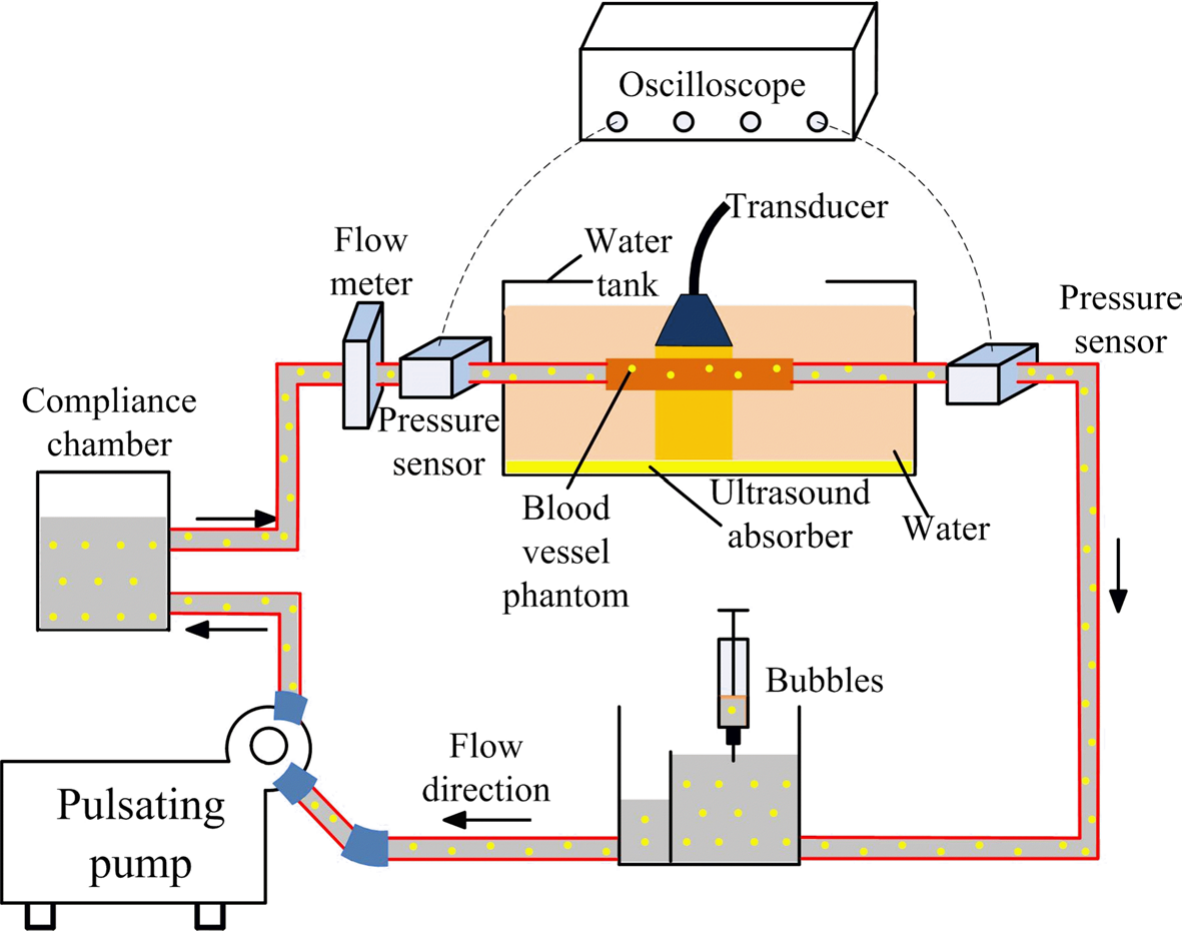
Experimental setup for Echo PIV. The pulsating flow was generated by a pump. Flow meter and pressure sensors connected to oscilloscope were used to record the boundary conditions which would be further used in CFD simulation. Phantoms were fixed in the circuit in water channel. Microbubbles with proper concentration were injected into circuit

**Fig. 3:**
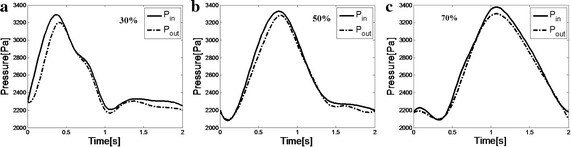
Averaged inlet and outlet flow pressure waveforms measured by pressure sensors during Echo PIV experiments for carotid phantoms with three severity, **a** 30%, **b** 50%, **c** 70%

The phantoms were placed in a flow loop as shown in Fig. 2, driven by a pulsatile blood pump (Hemodynamic Studies 553305, Harvard Apparatus, Holliston, USA), which can provide variable inlet flow rate and systolic/diastolic phase. A compliance chamber was placed between the pump and the phantom to serve as a cushioning application. The pulsation cycle in the study was 30 per minute. Care was taken to ensure that the tubing to the inlet and outlet was in the same horizontal level. In the study, the tube had 5 mm inner diameter and 1 mm wall thickness. According to the formula proposed by McDonald [38], to obtain a fully developed velocity profile in a 5 mm diameter tube with a mean velocity of 45 cm/s, the inlet length should be at least 70 cm. Two pressure transmitters (YB-131, Shanghai Zhengbao Meter Factory, Shanghai, China) were placed in the front and back of the phantom in combination with oscilloscope, so as to obtain the pressure waveforms (Fig. 3). Inlet flow rate was measured using a flow meter (LZB-10, Changzhou Chengfeng Flowmeter Co., Ltd, Jiangsu Prov., China). The working fluid was pure water with a nominal viscosity of 0.0087 Pa S under 26 °C. Ultrasound contrast microbubbles, which had a concentration of (5.0–8.0) × 10^8^ bubbles/ml as previously described in Niu et al. [39], were seeded into the working fluid as the imaging particles. The fluid ultrasonic image was obtained by vevo2100 with a MS250 transducer (40 MHz), which offered a frame rate of 150 fps, focal depth of 5 mm and field of view (FOV) of 7 mm (depth) by 15 mm (width), to allow for a full view of plaque.

### Ultrasonic particle image velocimetry algorithm

Echo PIV calculates particle displacements in a pair of particle images with traditional cross-correlation coefficient, using an interrogation window [24] firstly. Then sub-pixel method based on Gaussian peak fitting was adopted to estimate sub-pixel displacement [40, 41]. A local filter was used to manage spurious vectors which deviated from the mean of their surroundings. Lastly, a multiple iterative algorithm was adopted to deal with high velocity gradient flow and window deformation. According to our previous study [25], the images of the microbubble particles could be recorded with high-frequency ultrasound system. Analysis of the particle-images began with the manual selection of area-of-interest (ROI). The ROI was then analyzed on MATLAB R2014a using Echo PIV algorithm mentioned above. The interrogation window was set at 32 × 32 pixels with 50% overlap, in both the horizontal and vertical directions. 900 images (3 cycles) collected in one continuous acquisition were calculated during each operation. A formula as in (1) was used to estimate the SS through Echo PIV velocity distribution.

1 $$ \tau = \mu \left( {\frac{\partial v}{\partial x} + \frac{\partial u}{\partial y}} \right) $$

where $\mu$ is viscosity, $\frac{\partial v}{\partial x}$ velocity gradient along phantom wall, $\frac{\partial u}{\partial y}$ velocity gradient perpendicular to phantom wall.

### CFD simulation

CFD simulation in this study was performed using commercial FE solver ANSYS Workbench 15.0 (Ansys Inc, Canonsburg, PA, USA), which allows for the solution of Navier–Stokes (NS) equations with a finite volume approach in an ALE formulation as in (2, 3).

2 $$ \frac{\partial }{\partial t}\int_{V} {\rho dV} = \int_{S} {\rho (\overrightarrow {v} - \overrightarrow {w} )} \cdot \overrightarrow {n} dS $$

3 $$ \frac{\partial }{\partial t}\int_{V} {\rho \overrightarrow {v} } dV - \int_{S} {\rho \overrightarrow {v} } (\overrightarrow {v} - \overrightarrow {w} ) \cdot \overrightarrow {n} dS - \int_{S} \sigma \overrightarrow {n} dS = \int_{V} {\overrightarrow {f} } dV $$

where $V$ is the volume bounded by the surface $S$, $\overrightarrow {w}$ the velocity of the computational grid, $\rho$ the density of the fluid, $\overrightarrow {v}$ the fluid velocity and $\overrightarrow {n}$ the normal unit vector pointing out of the control volume, $\sigma$ the stress tensor and $\overrightarrow {f}$ the forces per unit of volume, pure water was modeled as a homogeneous and Newtonian fluid, the values of density was $\rho \;{ = }\; 1 0 0 0 \, \frac{\text kg}{{\text m^{2} }}$ and viscosity $\mu \;{ = }\; 0. 0 0 8 7\;{\text{Pa}}\;{\text{S}}$.

**Fig. 4:**
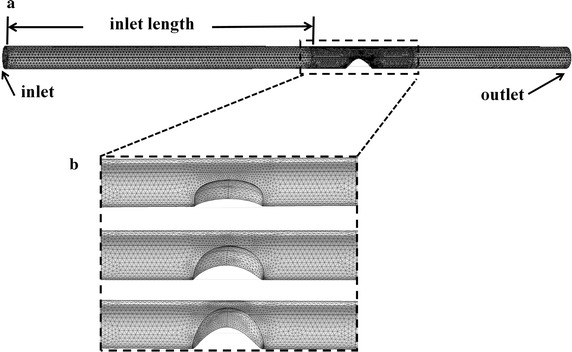
Meshes used in CFD simulation. All the geometries of three phantoms adopted triangle hexahedron grid type. **a** Full view of the meshes of phantoms; **b** detailed view of the meshes in carotid stenosis

Based on the assumption of Newtonian fluid and rigid wall, CFD was carried out by boundary condition measured during Echo PIV experiment as shown in Fig. 3. The CFD meshes for stenosis model were composed of triangle hexahedron elements as shown in Fig. 4. Table 1 shows the nodes and elements in three models.

**Table 1:** The nodes and elements in three models

Stenosis (%)	Nodes	Elements
30	36,228	107,960
50	34,660	100,162
70	32,913	92,534

## Results

### Velocity distributions measured by Echo PIV and CFD

**Fig. 5:**
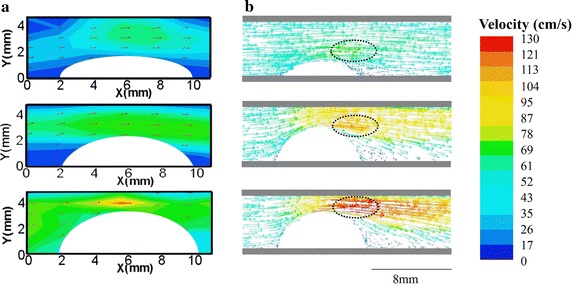
Velocity measured by Echo PIV and CFD in the systolic peak. **a** Velocity vectors measured by Echo PIV, *red arrows* represented the flow directors. An obvious vortex occurred in the back of 70% stenosis phantom. The peak blood velocity increased when increasing the stenosis degree. **b** Velocity vectors obtained by CFD. The *dotted portion* indicated the largest velocity areas

Figure 5 illustrates velocity distributions obtained by Echo PIV and CFD in central plane at the systole. Figure 5a indicates velocity vectors measured by Echo PIV, and the red arrows represent flow direction. The 30% stenosis had little effect on the flow pattern, and the peak velocity in the middle of the phantom was larger than that around the arterial wall. Due to Venturi effect, the velocity increased largely in the narrow parts of the phantoms in 50 and 70%. It was clear that the peak velocity in three phantoms (43.2, 69.7, 92.1 cm/s) enhanced when the stenosis degree of phantom increased. It could be observed that a 20% increase in stenosis resulted in nearly 50% increase in peak velocity. Figure 5b illustrates the systole peak velocity distributions obtained by CFD. The arrows represented the flow direction, and the color represented the velocity magnitude. Consistent with results measured by Echo PIV, the largest velocity occurred at the postmedian of the plaque (69.3, 95.7, 127.4 cm/s). We also made comparison for the velocity obtained by CFD and Echo PIV.

### Quantitative comparison of velocity distributions using CFD and Echo PIV

**Fig. 6:**
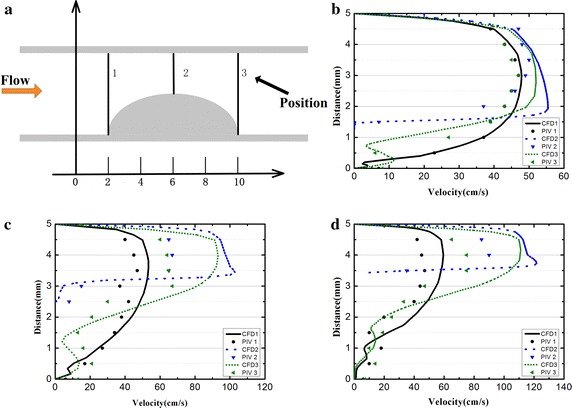
Quantitative comparison of velocity distribution calculated by CFD and Echo PIV. **a** The geometry of the stenotic phantom, the velocity vectors along the *three lines* were chosen to conduct comparisons between the measured values and the computationally simulated values; the velocity profiles along *three lines* in carotid phantoms with three severity, **b** 30%, **c** 50%, **d** 70%

A quantitative comparison of the velocity distributions obtained by CFD and Echo PIV in three stenosis phantoms. Figure 6a shows the geometry of the stenotic phantom. The velocity vectors along the three lines (marked as 1, 2, 3) were chosen to conduct comparisons between the measured values and the computationally simulated values. Figure 6 b–d illustrates the velocity profile obtained at the three positions of 30, 50, 70% stenotic phantoms. Lines with different colors represented CFD results, and dots with different shapes represented Echo PIV results. The peak velocity was 45.26 ± 6.12, 60.26 ± 5.36, and 89.11 ± 7.21 cm/s measured by Echo PIV in the carotid phantoms with different degrees of stenosis (30, 50, 70%), respectively. For the CFD results, the peak velocity was 57.04, 93.01 and 115.09 cm/s.

### WSS distributions measured by Echo PIV and CFD

**Fig. 7:**
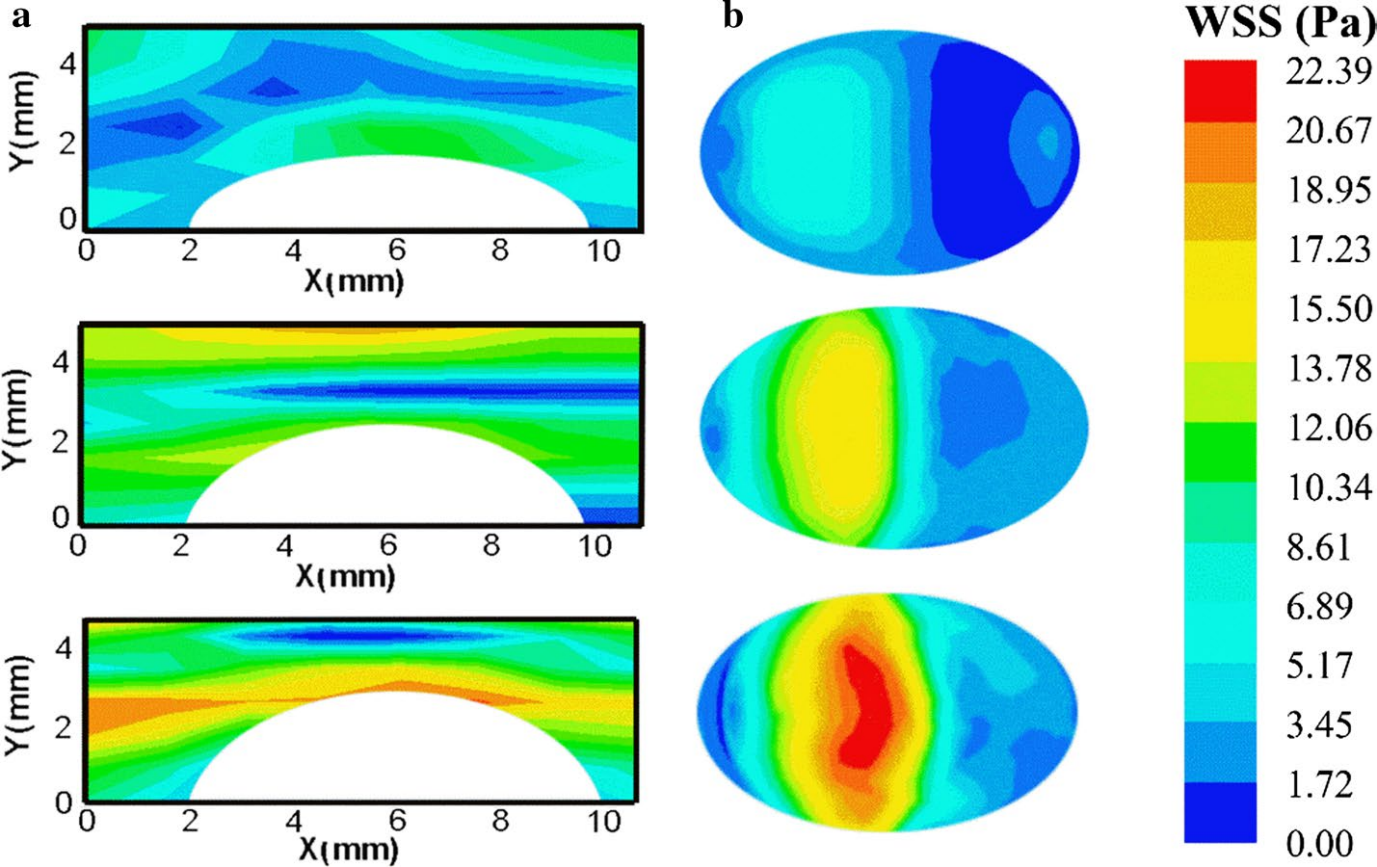
SS measured by Echo PIV and CFD in the systolic peak. **a** SS distribution in the central plane measured by Echo PIV in three stenosis phantoms. **b** WSS distribution in outer wall measured by CFD

Figure 7 shows the SS distributions obtained by Echo PIV in central plane and CFD in the surface of the plaque at the systole. Figure 7a indicates that in a stenosis phantom, high SS occurred in the upper wall and surface of plaque, and the head and shoulder bore larger WSS than other part of the plaque. Figure 7b shows the detailed WSS distributions on the surface of the plaque measured by CFD. It was obvious that the largest WSS occurred in the front of the plaque in all three stenosis phantoms, and WSS reached 22 Pa in the 70% stenosis. The largest WSS occurred in the downstream of the plaque calculated by Echo PIV, while it occurred in the upstream of the plaque measured by CFD. The largest WSS in the surface of plaque as showed in Table 2. It was clear that WSS measured by Echo PIV was less than CFD in the three phantoms.

**Table 2:** Largest WSS measured by Echo PIV and CFD in three stenosis

Stenosis (%)	Echo PIV (Pa)	CFD (Pa)
30	5	7
50	11	16
70	17	22

## Discussion

In this study, we conducted Echo PIV and CFD simulation in three different severity stenosis phantoms (30, 50, 70%), and further made comparisons on shear stress among three phantoms. Firstly, SS had functional relationship with velocity, and any changes in velocity would definitely cause corresponding changes in SS, thus we calculated the velocity profile by CFD and Echo PIV under the same condition. Both experiments demonstrated that there was a larger blood velocity changes in magnitude and direction in narrower phantom, which could result in abnormally high and low SS (Fig. 5). Secondly, we quantitatively calculated SS in three phantoms. Previous researches have indicated that SS can be used to understand the progress of atherosclerosis and may help to guide future therapeutic strategies [42]. Echo PIV and CFD results indicated that plaque shoulders generally exhibited high shear stress than other site. Previous studies have also indicated that plaque shoulders are most often the site of plaque rupture [43, 44]. Lastly, SS and velocity obtained from Echo PIV were obviously lower than those from CFD.

Several factors may contribute to different velocity results between CFD and Echo PIV. Firstly, CFD simulation simplified a rigid wall, whereas we used elastic phantoms in Echo PIV experiments. The effects of phantom elasticity, the resulting fluid–structure coupling and diameter expansions appeared to be obvious as shown in Table 3. To our knowledge, many researchers had a large interest in the reliability of CFD, and employed many tools, including MR, OCT, FSI [45–47], for the validation of CFD’s results. From the previous results, CFD can be widely used to calculate WSS and velocity, and resulting data is reliable in stiffness vessel [48]. However, the resulting data indicated a big discrepancy when the vessel wall is elastic. Many studies have indicated that SS and velocity calculated by CFD in elastic wall was larger than those by FSI [47]. It seems that the mechanical properties of vessel wall have large effect on shear stress. In our previous study, we calculated stress phase angle (SPA) in different arterial stiffness, and the results confirmed that different stiffness had different biomechanical parameters [49]. In addition, we speculated that the diameter expansions in stenosis domain may have a cushioning function on high speed fluid. Thus we observed a big disparity of velocity between CFD and Echo PIV in 50 and 70%.

**Table 3:** Diameter expansion in three stenosis during cardiac cycle

Stenosis (%)	Diameter expansion (mm)
30	5 ± 0.11
50	5 ± 0.06
70	5 ± 0.03

Secondly CFD relies on simplifying fluid conditions and specifies blood as Newtonian fluid (with constant viscosity respect to shear rate). However, blood exhibits non-Newtonian properties and variable shear-dependent viscosity. Previous studies indicated that different blood properties depicted different hemodynamic parameters [50, 51]. Finally, anatomically realistic artery stenosis model and vulnerable plaque model should be employed to further assess the probability of plaque rupture.

## Conclusion

In this study, we carried out CFD and Echo PIV analysis of hemodynamic shear stress in plaque phantoms with three severity stenosis phantoms. We observed that the degree of stenosis had a significant influence on the SS distribution, which was an important factor in the rupture of plaque. The results are a first step toward clinical application in prediction of plaque’s rupture. Future relevant work would include assessing the length of plaque influence on hemodynamic shear stress.
